# Plant exudates improve the mechanical conditions for root penetration through compacted soils

**DOI:** 10.1007/s11104-017-3424-5

**Published:** 2017-09-25

**Authors:** E. Oleghe, M. Naveed, E. M. Baggs, P. D. Hallett

**Affiliations:** 1 School of Biological Sciences, University of Aberdeen, Cruickshank Building, Aberdeen, AB24 3UU UK; 2 Department of Soil Science, Ambrose Alli University, P.M.B 14, Ekpoma, Edo State Nigeria; 3 The Royal (Dick) School of Veterinary Studies, University of Edinburgh, Easter Bush Campus, Midlothian, EH25 9RG UK

**Keywords:** Plant exudates, Void ratio, Cone penetration resistance, Compression index, Root growth modelling

## Abstract

**Background and aim:**

Plant exudates greatly affect the physical behaviour of soil, but measurements of the impact of exudates on compression characteristics are missing. Our aim is to provide these data and explore how plant exudates may enhance the restructuring of compacted soils following cycles of wetting and drying.

**Methods:**

Two soils were amended with Chia (*Salvia hispanica)* seed exudate at 5 concentrations, compacted in cores to 200 kPa stress (equivalent to tractor stress), equilibrated to −50 kPa matric potential, and then compacted to 600 kPa (equivalent to axial root stress) followed by 3 cycles of wetting and drying and recompression to 600 kPa at −50 kPa matric potential. Penetration resistance (PR), compression index (C_C_) and pore characteristics were measured at various steps.

**Results:**

PR decreased and C_C_ increased with increasing exudate concentration. At 600 kPa compression, 1.85 mg exudate g^−1^ soil increased C_C_ from 0.37 to 0.43 for sandy loam soil and from 0.50 to 0.54 for clay loam soil. After 3 wetting-drying cycles the clay loam was more resillient than the sandy loam soil, with resilience increasing with greater exudate concentration. Root growth modelled on PR data suggested plant exudates significantly eased root elongation in soil.

**Conclusion:**

Plant exudates improve compression characteristics of soils, easing penetration and enhancing recovery of root induced soil compaction.

## Introduction

Plant roots penetrate and alter the structure of compacted soils through the combined actions of exerting large radial and axial mechanical stresses, enhanced wetting and drying driven by evapotranspiration, as well as the release and secondary microbial decomposition of exudates (Watt et al. 2006; Hinsinger et al. 2009; Bengough et al. 2011; Gregory et al. 2013). They are so effective at improving soil physical conditions that biological tillage through the action of plant roots is a growing practice that is advocated in sustainable crop rotations. At the root-soil interface, the release of exudates by plant roots into the rhizosphere provides a major food source for microorganisms (Jones et al. 2004), induces a physico-chemical release of nutrients for plant uptake (Malamy 2005; Marco et al. 2015), and alters soil water retention and flow (Moradi et al. 2012; Zarebanadkouki and Carminati 2014). Whereas a large number of studies have explored biological and chemical properties of the rhizosphere, most physical investigations are limited to measures of soil stability or pore structure visualisation, as it is difficult to perform measurements at such a small scale (Peng et al. 2011; Czarnes et al. 2000; Morel et al. 1991).

A number of studies have adopted an approach of upscaling rhizosphere conditions by mixing plant exudate compounds with soil to form repacked samples that are large enough for measurements (Czarnes et al. 2000; Peng et al. 2011; Zhang et al. 2008). These have found a large impact of plant exudates on soil physical behaviour, which varies between plant species, seeds and roots. Exudates are often more viscous and have a lower surface tension than water (Read and Gregory 1997). This will have a large impact on the capacity of plants to capture water from soils. This was demonstrated by Carminati and Vetterlein (2013) and Carminati et al. (2010) who found that hydraulic conductivity and water uptake were enhanced by exudates after multiple cycles of wetting and drying. One driver is enhanced pore structure, which Reszkowska et al. (2011) found helped to recover hydraulic conductivity of rhizosphere soil in a degraded pasture field under wet conditions. Exudates can therefore decrease plant water stress by regulating water content dynamics and aiding capture of water in the rhizosphere (Kroener et al. 2014 and Ahmed et al. 2014).

Most of the studies mentioned above used model root exudates because real root exudates are difficult to extract and preserve in sufficient quantities The exudates have taken various forms, such as mucilages extracted from the seed coatings of *Salvia sp.* (Chia) (Kroener et al. 2014) or *Capsella sp.* (Deng et al. 2015). Major chemical components of root exudates, such as polygalacturonic acid (Czarnes et al. 2000), or biological exudates like xanthan produced by bacteria or scleroglucan produced by fungi, have also been used (Peng et al. 2011; Carminati and Vetterlein 2013; Carminati et al. 2010).

Physically, plant root growth induces pressure on soil particles (Misra et al. 1986). This pressure is compensated for by a loss in porosity resulting from a mechanically compressed zone of soil in the rhizosphere (Dexter 1987; Mooney et al. 2012). Plant root exudates influence root growth pressure and the porosity of the surrounding soil. Bengough and McKenzie (1997) described root exudates as a lubricant that decreases resistance arising from frictional contact between root surfaces and soil particles. Although many studies have examined the influence of plant root exudates on soil physical formation, there is a lack of information on how exudates impact compression characteristics of soil, which has a direct impact on root elongation and rhizosphere formation. A large challenge in this research is that the rhizosphere is physically small, so conventional soil compression tests are not feasible. To overcome this challenge we mixed soils of different texture with a range of concentrations of seed exudate from *Salvia hispanica*. Harvesting root exudates for such an experiment would be unfeasible due to the volumes required to form samples of adequate size. The soils were imparted with stresses to simulate vehicle compaction (200 kPa load), a growing plant root (600 kPa load) and recovery following cycles of wetting and drying. At each step of the experiment, porosity, water retention, penetration resistance and compression characteristics were quantified. All of these properties are known to influence hydrological and mechanical conditions for root growth and function. We hypothesised that plant exudates ease deformation by compression of soil, thereby creating a favourable condition for root growth where less energy needs to be exerted and stronger soils can be penetrated. With cycles of wetting and drying, we hypothesised that plant exudates would ease the impact of root induced soil compaction, thereby making the root-soil interface more resilient to this stress..

## Materials and methods

### Extraction of chia (*Salvia hispanica*) seed exudate

Chia seed exudate has been widely used in other studies as a model root exudate (Ahmed et al. 2014; Kroener et al. 2014). It was extracted based on Naveed et al. (2017) and Ahmed et al. (2014) by mixing 100 g distilled water with 10 g chia seeds using a magnetic stirrer for 2 min at 50 °C, followed by cooling to room temperature (20 °C) and 4 h standing. The exudate was separated from the seeds by repeatedly pushing the mixture through a 500 μm sieve under pressure using a syringe that was cut at the end. This approach harvested the easily extracted seed exudate, with tightly bound exudate remaining on the seeds even after 5 repeated extraction attempts. Of 0.13 ± 0.03 g g^−1^ (mean ± standard error) total exudate on seeds, only 0.10 ± 0.02 g g^−1^ of seed exudate was harvested, so the extraction efficiency was 77 ± 5%. The exudates were freeze-dried so that the dry weight of extracted chia seed exudate was 9.2 mg g^−1^ of the original exudate.

### Soil sampling, preparation of soil cores and mechanical measurements

Sandy loam and clay loam soils were sampled from the Ap horizon at the top 20 cm of Bullion field located at the James Hutton Institute, Dundee, UK), 56.27 N 3.40 W. The sandy loam soil is a Dystric Cambisol in arable production planted with barley, cultivated by ploughing to 20 cm depth. The clay loam soil is a Gleyic Cambisol, planted with deciduous trees, and was not mechanically cultivated. After sampling, bulk soils were air-dried, passed through a 2 mm sieve and stored in plastic bags at 4 °C before packing in soil cores. Both of the soils were treated with 0, 0.02, 0.2, 0.92 and 1.85 mg g^−1^ concentrations of chia seed exudates, wetting the soils to 0.20 g g^−1^ gravimetric water content. These treated soils were stored in sealed plastic bags at 4 °C for 15 days to allow equilibration of samples with minimal microbial decomposition.

**Fig. 1:**
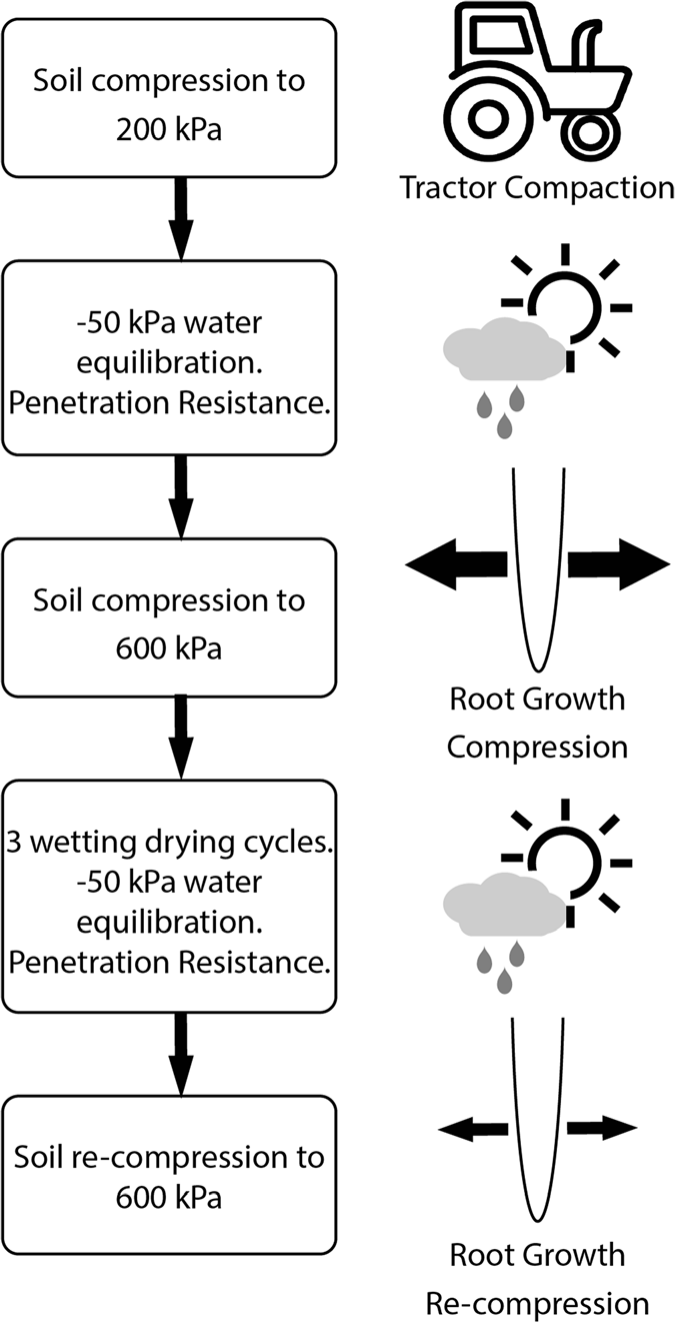
Flow chart of the experimental programme; 200 kPa stress was simulated as vehicle traffic, 600 kPa compression stress was simulated as stress induced by a growing root in the soil and 3 wetting-drying cyles were simulated as natural weathering at the root-soil interface

The flow chart of the experimental programme is shown in Fig. 1. There were three different steps in forming and conditioning the soil samples: (i) 200 kPa loading, (ii) 600 kPa loading and (iii) 600 kPa loading with wetting and drying.

Forty grams of treated soils at 0.20 g g^−1^ gravimetric water content were packed in 0.5 cm layers into plastic cores (H = 2 cm, D = 5 cm) with a compression plate to a stress of 2.5 kPa. This produced samples with an initial bulk density of 1.0 g cm^−3^ and produced a flat upper surface to provide accurate displacement measurements during compression testing. Five replicates of each treatment were formed. Soil cores were then equilibrated to −50 kPa water potential and conditioned to simulate vehicle compaction by compressing to 200 kPa with a mechanical test frame (Zwick All Round Z5, Zwick-Roell, Ulm, Germany) fitted with a 5 kN load cell. It took 5 min to reach 200 kPa. Data on applied stress and displacement were captured to evaluate compression characteristics. After that, soil cores were saturated for 12 h and dried until water loss ceased (2–3 days) to −50 kPa matric potential using a tension table (EcoTech MeBaystem GmbH, Germany) at 4 °C to minimise microbial decomposition. Cone penetration tests and confined compression tests were then performed. Penetration resistance (PR) was measured using a 1 mm diameter, 30^o^ full opening angle miniature penetrometer tip attached to a 5 kN load cell using the mechanical test frame described previously. The cone was inserted to a depth of 4 mm at a speed of 2 mm/min. One cone penetration test was carried out per soil core to minimise damage before confined compression tests. Confined compression tests to 600 kPa were performed on the same soil cores to exert a similar stress to a growing root (Misra et al. 1986). The loading rate to simulate root growth through soil took 20 min to reach 600 kPa. Mean values of the maximum axial root growth pressure estimated from the maximum axial root growth force and root diameter are 497, 289, and 238 kPa respectively for pea, cotton and sunflower seedlings (Misra et al. 1986). After this, these compressed soil cores were equilibrated to −50 kPa matric potential on a tension table at 4 °C. Three cycles of wetting and drying from saturation to −50 kPa matric potential were then imposed to simulate natural weathering, followed by compression again at 600 kPa stress and −50 kPa matric potential.

### Analysis of data

**Fig. 2:**
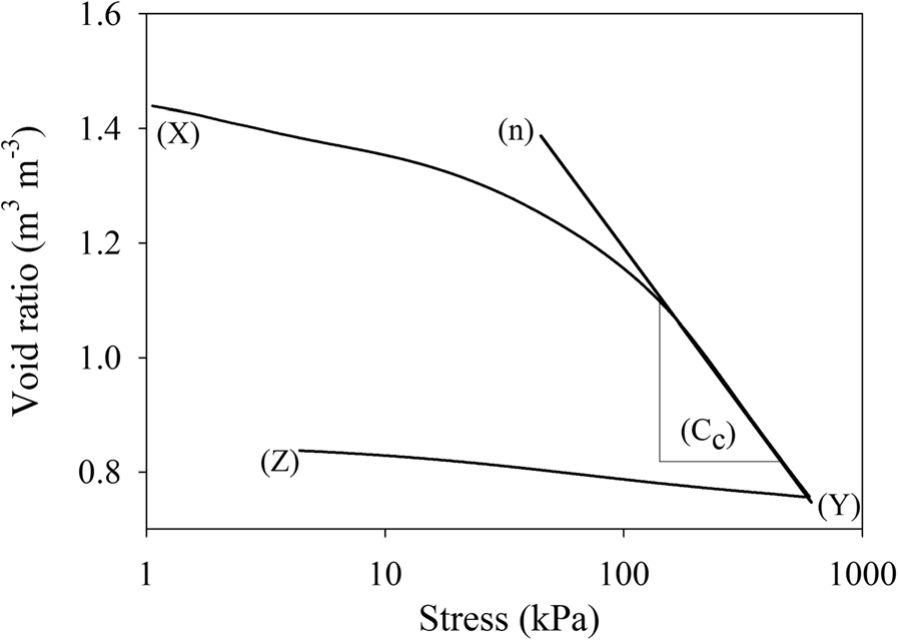
Interpretation of confined compression tests showing loading for the soil from (X) to (Y), followed by unloading from (Y) to (Z). Data are plotted as void ratio as a function of log_10_ stress, with compression index (C_C_) calculated as the slope of the virgin compression curve

PR data were expressed as cone penetration resistance (MPa). The confined compression tests data were plotted as Log_10_ stress (kPa) as a function of void ratio (cm^3^ cm^−3^) and a virgin compression curve was obtained. The slope of the virgin compression curve is commonly called the compression index (C_C_), which was calculated as shown in Fig. 2. In addition to this, three other parameters i.e. void ratio (total porosity to the volume of soil solids), air ratio (air-filled porosity to the volume of soil solids) and water ratio (volumetric water content to the volume of soil solids) were calculated from soil core weights and volumes.

Root growth for maize at −50 kPa matric potential was modelled based on PR data using Dexter’s (1987) model given as Eq. 1.

1 $$ \frac{R}{R_{\mathrm{max}}}=\frac{\psi_0}{\psi_{\mathrm{w}}}+{\mathrm{e}}^{-0.6931\left(\frac{Q_{\mathrm{p}}}{Q_{1/2}}\right)} $$

where *R* is rate of root elongation (mm day^−1^), *R*_max_ maximum rate of maize root elongation of 26 mm day^−1^ (Mirreh and Ketcheson 1973), *ψ*_0_ is water potential in MPa, *ψ*_w_ is the wilting point water potential i.e. -1.5 MPa, *Q*_*P*_ is the cone penetration in MPa and *Q*_1/2_ is the cone penetration resistance that reduces relative root elongation rate to one-half (taken as 1.3 MPa for maize).

### Statistical analysis

The experiment was setup as a Completely Randomised Design (CRD) with 5 levels of added exudates, 2 soil textures and 5 replicates. Exponential, log or linear models were selected to fit the measured data based on their fitting efficiency i.e. random distribution of model residuals as a function of dependent variable and higher *R* ^2^ value. The significant difference between individual exudate treatments was tested using one way analysis of variance (ANOVA). To test the effect of exudate concentration and loading conditions as a whole on compression index, cone penetration resistance and root elongation rate, analysis of covariance (ANCOVA) was carried out using SigmaPlot 13. In ANCOVA, compression index, cone penetration resistance and root elongation rate as response variables, exudate concentration as covariates and different loading conditions as factors were used. Bonferroni t-test was used for all pairwise comparisons at *P* < 0.05. A summary of ANCOVA for exudate concentration and loading condition was provided.

## Results

### Exudate and soil properties

The chia seed exudate consisted of 40.7 g 100 g^−1^ carbon, 1.1 g 100 g^−1^ nitrogen and the carbon nitrogen ratio was 37. It had a pH-H_2_O of 6.9 at 9.2 mg g^−1^ concentration. The physical properties of the studied soils are shown in Table 1. The soil texture was sandy loam for the soil sampled from south Bullion and clay loam for the soil sampled from north Bullion. Total carbon content for the sandy loam soil was 2.25 g 100 g^−1^ and for clay loam soil was 2.95 g 100 g^−1^. The soil pH_Cacl_2_ at 1:5 soil to water was 5.48 for sandy loam soil and 5.15 for the clay loam soil (Table 1).

**Table 1:** Characteristics of the soils

Location	Clay	Silt	Sand	Carbon	Nitrogen	Soil pH_Cacl_2_	Texture class
	(g. 100 g^−1^)						
South Bullion	16	24	60	2.25 ± 0.14	0.16 ± 0.03	5.48 ± 0.07	Sandy loam
North Bullion	26	30	44	2.95 ± 0.12	0.23 ± 0.02	5.15 ± 0.04	Clay loam

Mean ± s.e.m. of 3 replicates

### Plant exudate impact on soil compression characterisitcs

**Fig. 3:**
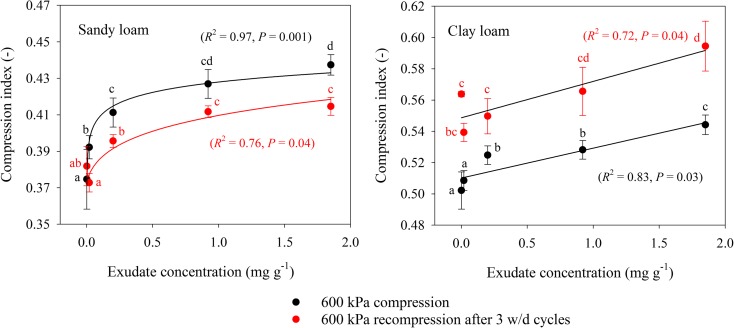
Compression index at −50 kPa matric potential plotted as a function of exudate concentration for sandy loam and clay loam soils for (i) 200 kPa loading, (ii) 600 kPa loading and (iii) 600 kPa loading with wetting and drying. Error bars represent ±1 s.e.m. (*n* = 5). Different lowercase letters show a significant difference (*P* < 0.05) between either exudate concentration or stages of the compression cycle

A summary of statistical analyses is provided in Table 2. The compression index (C_C_) measures soil mechanical resistance to compression, with larger values indicating less resistance of soil to compression. C_C_ for 600 kPa compression increased by 17% for the sandy loam soil and 9% for the clay loam soil between 0 and 1.85 mg g^−1^ exudate amendment (Fig. 3). Three cycles of wetting and drying, followed by recompression to 600 kPa had contrasting effects on C_C_ between soils. Both soils followed the same trend with increasing exudate concentration as observed for the soils before wetting and drying, but C_C_ had an overall drop of 5% for the sandy loam soil and increased by 7% for the clay loam soil (*P* < 0.001).

**Table 2:** Summary of analysis of covariance (ANCOVA) for different parameters

Source	*df*	*SS*	*MS*	*F*	*P*
Compression index, Sandy loam					
Exudate concentration	1	0.003	0.003	19.99	0.003
Loading condition	1	0.0004	0.0004	2.98	0.128
Residual	7	0.001	0.0002		
Total	9	0.0043	0.0005		
Compression index, Clay loam					
Exudate concentration	1	0.002	0.002	51.3	<0.001
Loading condition	1	0.004	0.004	27.7	0.001
Residual	7	0.0006	0.0001		
Total	9	0.007	0.0008		
Cone penetration resistance, Sandy loam					
Exudate concentration	1	0.116	0.116	6.09	0.043
Loading condition	1	0.11	0.11	5.76	0.047
Residual	7	0.133	0.019		
Total	9	0.359	0.039		
Cone penetration resistance, Clay loam					
Exudate concentration	1	0.006	0.006	7.72	0.027
Loading condition	1	0.028	0.028	36.7	<0.001
Residual	7	0.0054	0.001		
Total	9	0.039	0.004		
Root elongation rate, Sandy loam					
Exudate concentration	1	14.47	14.47	6.52	0.038
Loading condition	1	14.45	14.45	6.51	0.038
Residual	7	15.53	2.22		
Total	9	44.45	4.94		
Root elongation rate, Clay loam					
Exudate concentration	1	1.03	1.03	8.13	0.025
Loading condition	1	4.89	4.89	38.67	<0.001
Residual	7	0.89	0.13		
Total	9	6.81	0.76		

*df*, degree of freedom; *SS*, sum of squares; *MS*, mean squares

### Pore characteristics

**Fig. 4:**
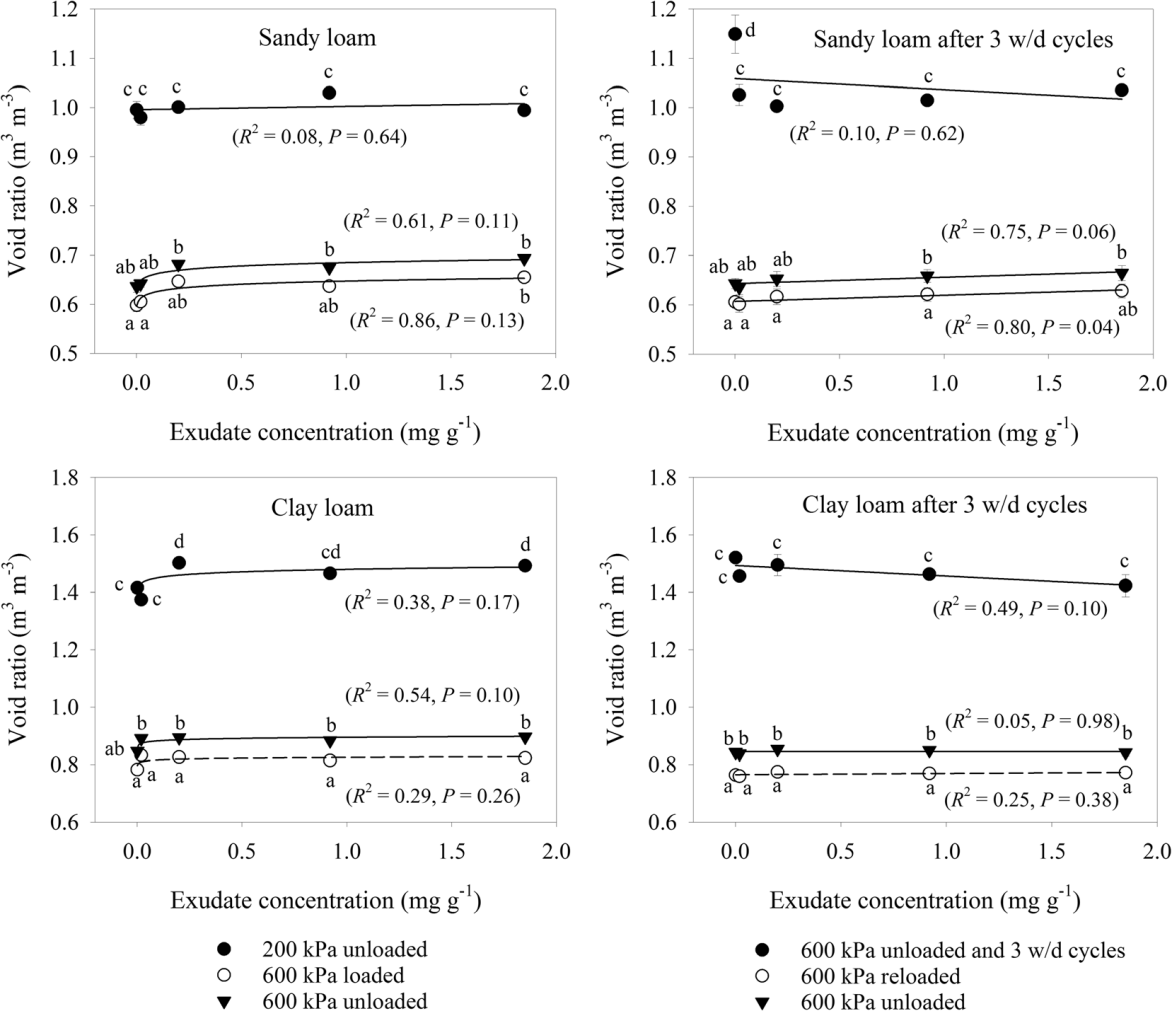
Void ratio relationship to exudate concentration for sandy loam and clay loam soils for (i) 200 kPa loading, (ii) 600 kPa loading and (iii) 600 kPa loading with wetting and drying. Error bars represent ±1 s.e.m. (*n* = 5). Different lowercase letters show a significant difference (*P* < 0.05) between either exudate concentration or stages of the compression cycle

After 200 kPa compression, there was no relationship between exudate concentration and void ratio for either soil (Fig. 3), although for the clay loam soil there was an increase in void ratio for any of the exudate amendent levels compared to the control (*P* < 0.05). Further compression to 600 kPa stress resulted in a drop in void ratio of at least 0.30 m^3^ m^−3^ for the sandy loam soil and 0.50 m^3^ m^−3^ for the clay loam soil, with both soils rebounding in void ratio by about 0.05 m^3^ m^−3^ after the compression stress was removed. Under 600 kPa compression and rebound, any exudate amendment level had greater void ratio than the control (*P* < 0.05) for both soils, with a significant relationship between exudate concentration and void ratio found only for the sandy loam soil (Fig. 4).

There was a marked recovery in void ratio of the 600 kPa compressed soils after 3 wetting-drying cycles, but no influence of exudate amendment apart from greater recovery of the 0 mg g^−1^ exudate control for the sandy loam soil (Fig. 4). ANCOVA analysis found recovery was close to the initial conditions before the 600 kPa stress had been applied (*P* > 0.05). Moreover, re-compression characteristics were also similar to the initial 600 kPa loading, with exudate concentration having a positive correlation with void ratio under loading and unloading conditions only for the sandy loam soil.

**Fig. 5:**
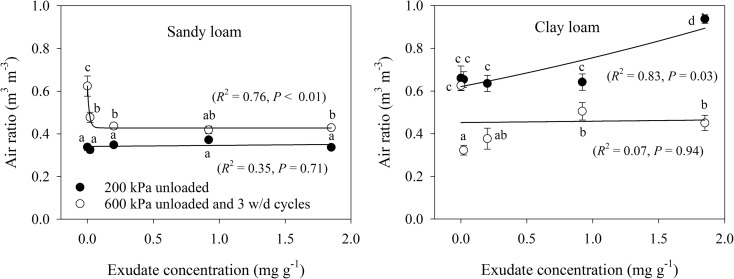
Air ratio relationship to exudate concentration for sandy loam and clay loam soils for (i) 200 kPa loading, (ii) 600 kPa loading and (iii) 600 kPa loading with wetting and drying. Error bars represent ±1 s.e.m. (*n* = 5). Different lowercase letters show a significant difference (*P* < 0.05) between either exudate concentration or stages of the compression cycle

**Fig. 6:**
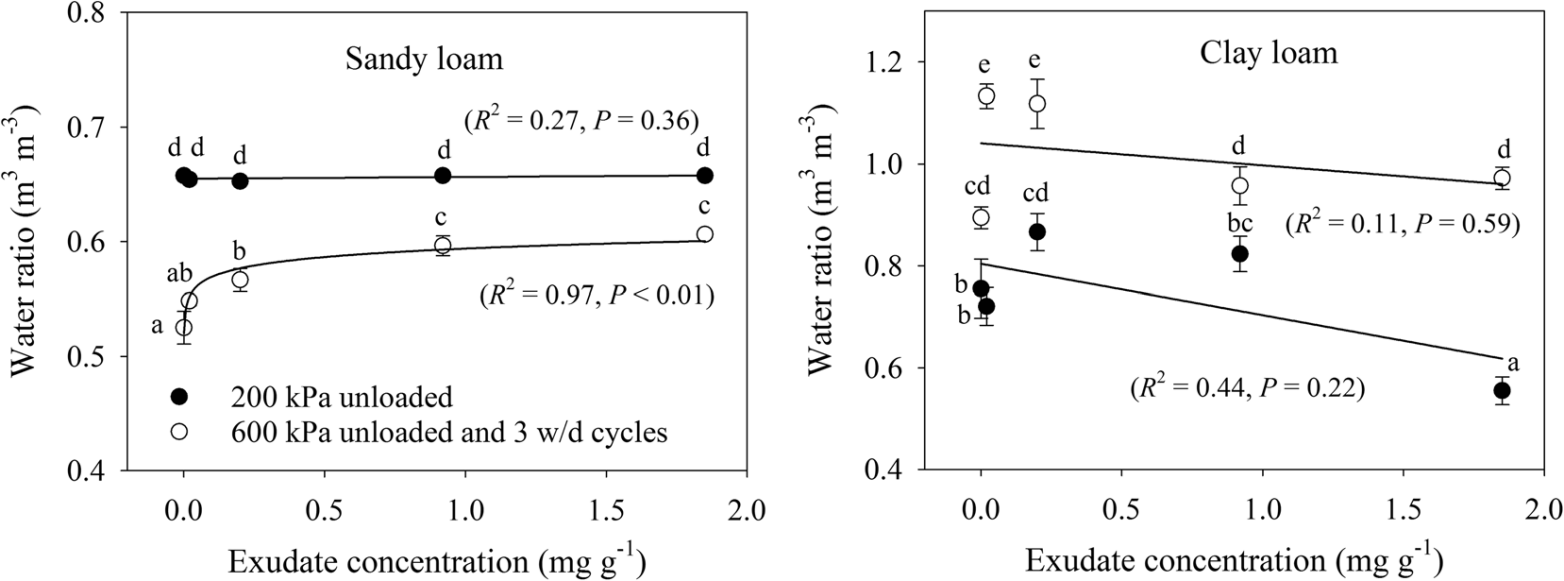
Water ratio relationship to exudate concentration for sandy loam and clay loam soils for (i) 200 kPa loading, (ii) 600 kPa loading and (iii) 600 kPa loading with wetting and drying. Error bars represent ±1 s.e.m. (*n* = 5). Different lowercase letters show a significant difference (*P* < 0.05) between either exudate concentration or stages of the compression cycle

Void ratio consists of a water and air phase, which are expressed as air and water ratios in Figs. 5 and 6, respectively. The data illustrate the expected trend of increasing air ratio with decreasing water ratio, and vice versa. In the sandy loam soil, there was no effect of exudate concentration on either air or water ratio after 200 kPa compression, but following 600 kPa compression and 3 cycles of wetting and drying, increasing exudate concentration decreased air ratio and increased water ratio. The sandy loam samples after 600 kPa compression and 3 cycles of wetting and drying had more air and less water, which was the opposite of the clay loam soil and verified with ANCOVA analysis (*P* < 0.05). The only relationship found for the clay loam soil was increasing air ratio with increasing exudate concentration. There were minimal, but statistically significant differences between pairs of exudate concentrations, but the trends were erratic for the other measurements.

### Penetration resistance and modelled root growth

**Fig. 7:**
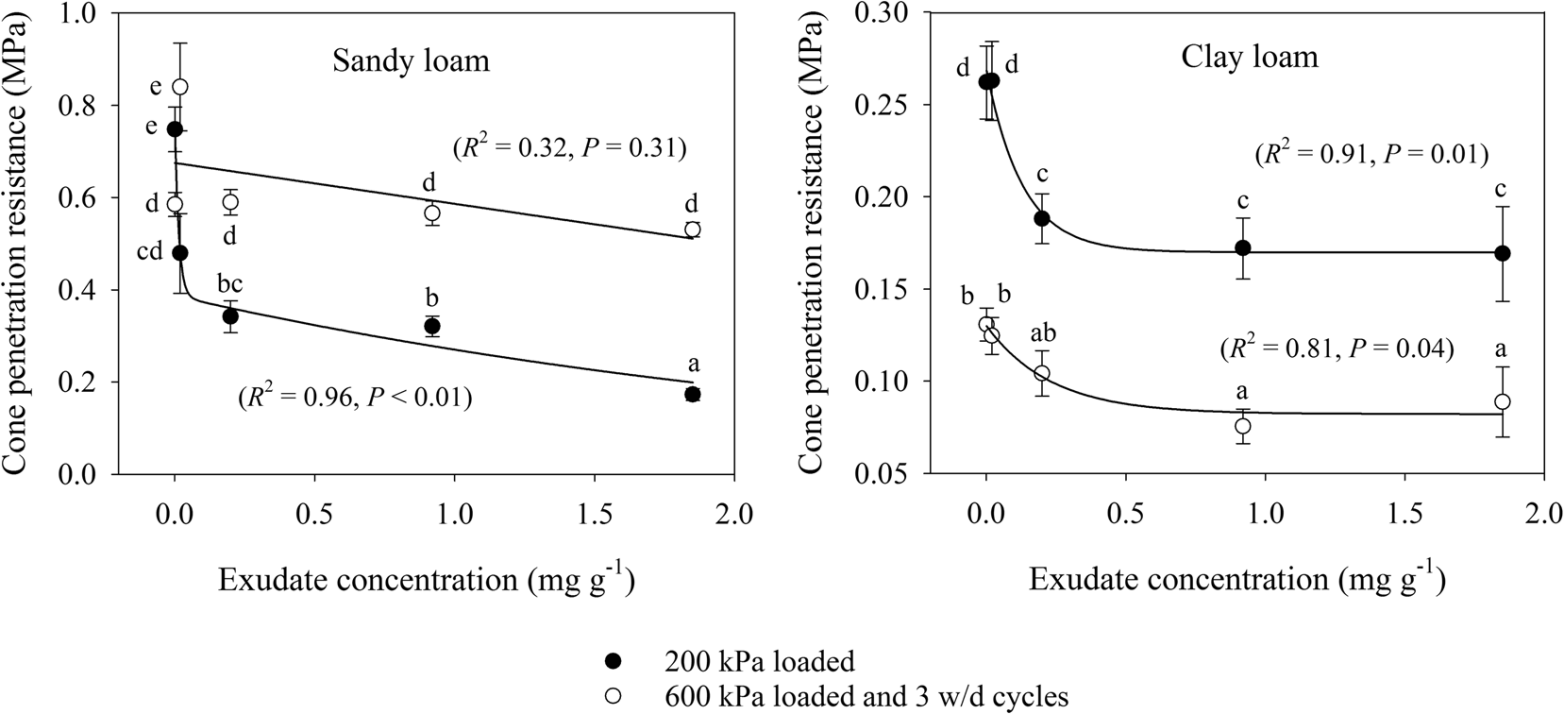
Cone penetration resistance at −50 kPa matric potential relationship to exudate concentration for sandy loam and clay loam soils for (i) 200 kPa loading, (ii) 600 kPa loading and (iii) 600 kPa loading with wetting and drying. Error bars represent ±1 s.e.m. (*n* = 5). Different lowercase letters show a significant difference (*P* < 0.05) between either exudate concentration or stages of the compression cycle

The two different stages of PR measurements illustrated in Fig. 7 are for conditions immediately after compression by a 200 kPa stress to simulate vehicle traffic, and after 600 kPa with three cycles of gentle wetting and drying, to simulate a compressed region of soil around a root after weathering. For 200 kPa compression, increasing the amount of exudate from 0 to 1.85 mg g^−1^ decreased PR by 77% for the sandy loam soil and 36% for the clay loam soil, demonstrating that exudates ease penetration into compacted soils. In the simulated root zone, with 600 kPa stress and 3 cycles of wetting and drying, the same exudate amendment had less of an effect on the sandy loam soil, with only a 10% decrease, whereas it was 32% for the clay loam soil. ANCOVA showed that PR between the 200 kPa and 600 kPa with wetting and drying treatments increased for the sandy loam soil and decreased for the clay loam soil (*P* < 0.001).

**Fig. 8:**
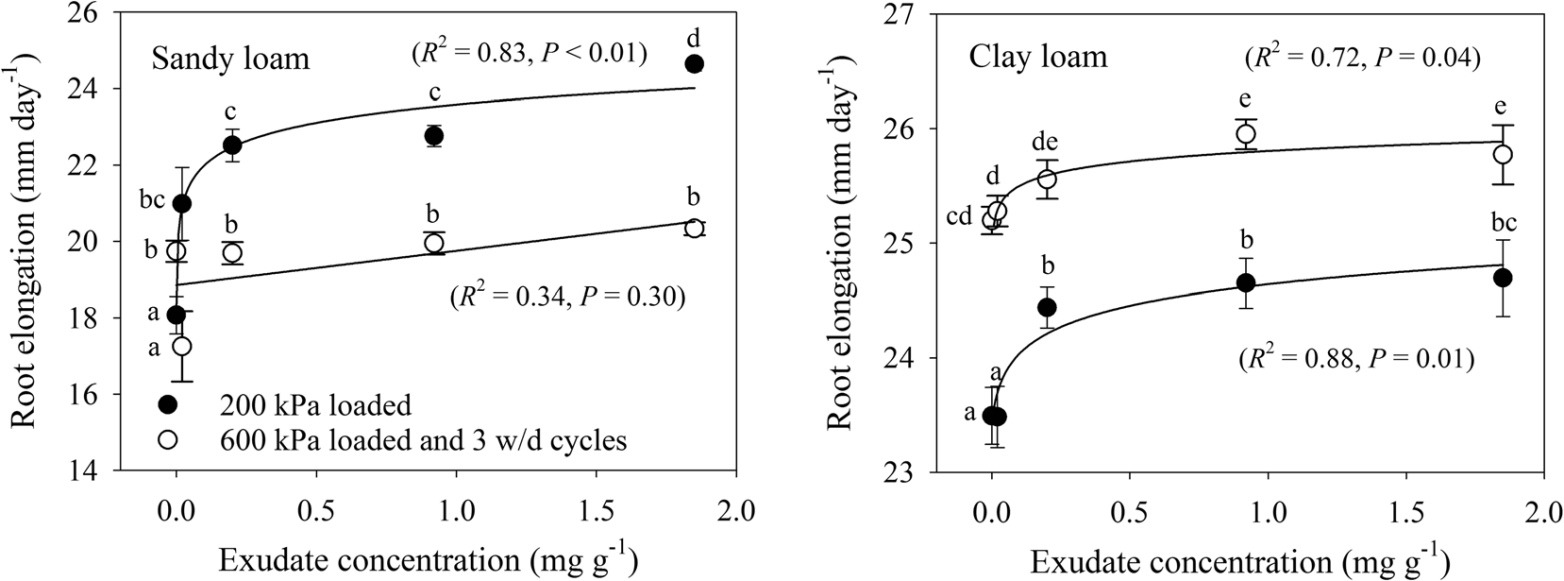
Modelled root elongation rate using Dexter’s (1987) model at −50 kPa matric potential for sandy loam and clay loam soils for (i) 200 kPa loading, (ii) 600 kPa loading and (iii) 600 kPa loading with wetting and drying. Error bars represent ±1 s.e.m. (*n* = 5). Different lowercase letters show a significant difference (*P* < 0.05) between either exudate concentration or stages of the compression cycle

Based on Dexter’s (1987) root growth model, which uses penetration resistance to describe the mechanical condition of the soil, we calculated that the root elongation rate (mm day^−1^) increased markedly with increasing exudate concentration (Fig. 8). For the sandy loam soil, the increase was over 30%, but subsequent cycles of wetting and drying diminished the positive impact of the exudates. Root elongation rate in the clay loam only increased by about 5%, with cycles of wetting and drying causing a further increase.

## Discussion

Plant exudates obtained from *Salvia hispanica* seed coatings were found to greatly improve mechanical conditions for root growth, quantified from compression characteristics and penetration resistance. The decrease in penetration resistance of both sandy loam and clay loam soils with increasing exudate concentration (Fig. 7) demonstrates that exudates decrease soil resistance to local deformation. Similarly, an increase in compression index for both sandy loam and clay loam soils with increasing exudate concentration (Fig. 3) means the exudate used eased soil compression. We have not found any study in the literature reporting compressibility of soil treated with plant exudates. There are several studies reporting the impact of organic matter on soil compressibility that are useful to interpreting our results. Ekwue et al. (2014) reported a considerable decrease in shear strength and cone penetration resistance for loam and clay soils with increasing organic matter contents. Stock and Downes (2008) found a decrease in cone penetration resistance with increasing soil organic matter and water content of a glacial till. Similarly Zhang et al. (2005) measured increased soil compressibility with added particulate organic matter amendment, which is consistent with our hypothesis.

In addition to easing mechanical conditions for root growth, exudates also enhanced the resilience of soil to a 600 kPa compression used to simulate axial root growth stresses. After 3 cycles of wetting and drying, increasing exudate concentration decreased the penetration resistance and increased the compression index (Figs. 7 and 3). For a growing root that is transpiring water from soil, this improved resilience in the presence of exudates indicates potential structural re-arrangement of rhizosphere soil over time, creating better physical conditions for root elongation. Field based evidence of the capacity of plant roots to enhance mechanical resilience of soil was provided by Gregory et al. (2007), who found penetration resistance of a compacted soil to decrease far more in the presence of roots than in fallow soil in a sandy loam soil. The capacity of plant roots to restructure compacted soils is well reported (Uteau et al. 2013; Bodner et al. 2014), driven by a combination of roots fracturing soil, enhancing cycles of wetting and drying, producing biopores and secreting exudates (Gregory et al. 2013; Materechera et al. 1992).

The possible mechanisms driving the changes in compression behaviour of soil as a result of exudation could be the amount of water retained by the exudates (Carminati et al. 2011) and hence effective stress, a lubricating effect of exudates that may decrease interparticle friction (Bengough et al. 2011) and the role of exudates in the dispersion, aggregation and hence pore structure development of soil (Deng et al. 2013). A more porous soil would be expected to be more compressible, but pore structure interactions with soil mechanical behaviour were not found for the sandy loam soil that we studied. After 600 kPa stress, none of void ratio, air ratio or water ratio for the sandy loam soil were correlated with penetration resistance or compression index. However, the clay loam soil after 600 kPa stress had a positive correlation between void ratio and compression index, and a negative correlation between void ratio penetration resistance. In this soil a more open pore structure therefore had the expected impact of decreased mechanical resistance. As pore structure did not influence the compression characteristics of the sandy loam soil, a lubricating effect of exudates was possibly the major driver.

After 600 kPa compaction stress followed by 3 cycles of wetting and drying, the relationships of void ratio, water ratio or air ratio with compression index or penetration resistance were more erratic. Penetration resistance was not correlated with any of these pore properties for either soil. The correlation between water ratio and the compression index of the sandy loam soil suggests exudate driven retention of pore water to influence mechanical behaviour. However, the same trend was not observed for the clay loam soil, probably due to clay dominating over exudates in water retention (Fig. 6). Compression index tends to increase for soils with greater clay content due to greater plasticity and void ratios (Gregory et al. 2006). Moreover, the clay loam soil had greater resilience to compression (Figs. 4, 5, 6, 7, and 8) due to the shrink-swell nature of clays and possibly the slightly greater organic carbon content compared to the sandy loam soil (Gregory et al. 2007). This mechanical resilience was reflected in the penetration resistance (Fig. 7) and modelled root elongation rate (Fig. 8), where 3 cycles of wetting and drying can weaken a soil compressed to 600 kPa to less than it was at 200 kPa loading. Interestingly, few of the measures of pore structure in either the sandy loam or clay loam soil were responsive to exudate amendment, but the mechanical measurements were very responsive (Figs. 3 and 7). The mechanical conditions of structured soils are driven by a myriad of processes, so simple relationships with bulk pore structure or water retention should not be expected (Keller et al. 2013), even for a model system that begins with homogenised soils, simple biological amendments and controlled drying and wetting.

Although exudation clearly represents a significant carbon cost to the plant, exudates are involved in engineering the rhizosphere by dispersion and gelling of soil (Naveed et al. 2017; Barré and Hallett 2009; Tarchitzky and Chen 2002; Deng et al. 2015), modulation of water and nutrient availabilities (Wang et al. 2008; Ahmed et al. 2014; Deng et al. 2015), and attraction of rhizobacteria (Bais et al. 2006). To our knowledge this is the first time that plant exudates have been demonstrated to ease soil compression and thus offer the potential for increased root elongation in soil. This could have remarkable effects on overall plant growth as it will influence the capacity of roots to access deep and disperse water and nutrient resources in soil. In structured soils roots prefer to follow pathways of least resistance (Landl et al. 2017), with evidence of attraction of roots towards macropores where mechanical impedance will be much smaller (Colombi et al. 2017). However, macropore networks are discontinuous so roots need to penetrate bulk soil to reach them. Good root:soil contact is also required for resource capture (Schmidt et al. 2012), which is poorer in macropores and could be enhanced by localised changes in mechanical conditions of surrounding soil by root exudates.

We appreciate that using chia seed exudate as a model root exudate has limitations. A recent study by Naveed et al. (2017) found that chia seed exudate has a greater amount of polysaccharide sugars and less organic acids than barley and maize root exudates, with differing impacts on soil rheology and water retention. Given the scale of samples required for compression experiments, however, harvesting real root exudates in sufficient quantities would be a formidable task. Whilst future research could explore impacts of real root exudates, model root exudate compounds formed from mixes of sugars and amino acids (e.g. Paterson et al. 2007) would allow for the impact of specific chemical characteristics to be disentangled. Such information will be useful in selecting plant species or in identifying root exudate biochemical traits in breeding that could have positive physical impacts on soil.

## Conclusions

Plant exudates eased soil compression and improved the mechanical resilience of compacted soils; the latter possibly having a large positive impact on rhizosphere physical conditions. The modelled increases in root elongation rate in soil, which was 40% faster in the sandy loam than the clay loam, are likely to impact on the capacity of roots to explore deep and disperse soil regions for resources. The physically quantified data generated from this study will be useful for models of how plant exudates may influence root growth and impact soil pore structure. Future research with model root exudates that vary in chemistry, real root exudates and plants with contrasting exudation properties could identify favourable exudate characteristics that improve the capacity of roots to grow in and restructure degraded soils. Such understanding would benefit practical applications of biological tillage by plants, selecting species in crop rotations to improve soil physical conditions and in crop breeding to improve the capacity of roots to grow through and restructure soils.

## References

[CR1] Ahmed M, Kroener E, Maire H, Mohsen Z, Carminati A (2014). Mucilage exudation facilitates root water uptake in dry soils. Funct Plant Bio.

[CR2] Bais HP, Weir TL, Perry LG, Gilroy S, Vivanco JM (2006). The role of root exudates in rhizosphere interactions with plants and other microorganisms. Annu Rev Plant Bio.

[CR3] Barré P, Hallett PD (2009). Rheological stabilization of wet soils by model root and fungal exudates depends on clay mineralogy. Eur J Soil Sci.

[CR4] Bengough AG, McKenzie BM, Hallett PD, Valentine TA (2011). Root elongation, water stress, and mechanical impedance: a review of limiting stresses and beneficial root tip traits. J Exp Bot.

[CR5] Bengough AG, Mckenzie BM (1997). Sloughing of root cap cells decreases the frictional resistance to maize (L.) root growth. J Exp Bot.

[CR6] Bodner G, Leitner D, Kaul HP (2014). Coarse and fine root plants affect pore size distributions differently. Plant Soil.

[CR7] Carminati A, Vetterlein D (2013). Plasticity of rhizosphere hydraulic properties as a key for efficient utilization of scarce resources. Ann Bot.

[CR8] Carminati A, Schneider CL, Moradi AB, Zarebanadkouki M, Vetterlein D, Vogel HJ, Hildebrandt A, Weller U, Schuler L, Oswald SE (2011). How the rhizosphere may favor water availability to roots. Vadose Zone J.

[CR9] Carminati A, Moradi A, Vetterlein D, Vontobel P, Lehmann E, Weller U (2010). Dynamics of soil water content in the rhizosphere. Plant Soil.

[CR10] Colombi T, Braun S, Keller T, Walter A (2017). Artificial macropores attract crop roots and enhance plant productivity on compacted soils. Sci Tot Env.

[CR11] Czarnes S, Hallett PD, Bengough AG, Young IM (2000). Root- and microbial- derived mucilages affect soil structure and water transport. Eur J Soil Sci.

[CR12] Deng W, Hallett PD, Jeng D-S, Squire GR, Toorop PE, Iannetta PPM (2015). The effect of natural seed coatings of Capsella bursa-pastoris L. Medik. (shepherd’s purse) on soil-water retention, stability and hydraulic conductivity. Plant Soil.

[CR13] Deng W, Iannetta PPM, Hallett PD, Toorop PE, Squire GR, Jeng D-S (2013). The rheological properties of the seed coat mucilage of *Capsella bursa-pastoris* L. Medik. (shepherd's purse). Biorheology.

[CR14] Dexter AR (1987). Mechanics of root growth. Plant Soil.

[CR15] Ekwue EI, Birch RA, Chadee NR (2014). A comparison of four instruments for measuring the effects of organic matter on the strength of compacted agricultural soils. Biosyst Eng.

[CR16] Gregory PJ, Bengough AG, George TS, Hallett PD (2013) Rhizosphere engineering by plants: quantifying soil–root interactions. In: Timlin D and Ahuja L (eds) Enhancing understanding and quantification of soil–root growth interactions, 1–30

[CR17] Gregory AS, Watts CW, Whalley WR, Kuan HL, Griffiths BS, Hallett PD, Whitmore AP (2007). Physical resilience of soil to field compaction and the interactions with plant growth and microbial community structure. Eur J Soil Sci.

[CR18] Gregory AS, Whalley WR, Watts CW, Bird NRA, Hallett PD, Whitmore AP (2006). Calculation of the compression index and precompression stress from soil compression test data. Soil Till Res.

[CR19] Hinsinger P, Bengough AG, Vetterlein D, Young IM (2009). Rhizosphere: biophysics, biogeochemistry and ecological relevance. Plant Soil.

[CR20] Jones DL, Hodge A, Kuzyakov Y (2004). Plant and mycorrhizal regulation of rhizodeposition. New Phytol.

[CR21] Keller T, Lamande M, Peth S, Berli M, Delenne JY, Baumgarten W, Rabbel W, Radjai F, Rajchenbach J, Selvadurai APS, Or D (2013). An interdisciplinary approach towards improved understanding of soil deformation during compaction. Soil Till Res.

[CR22] Kroener E, Zarebanadkouki M, Kaestner A, Carminati A (2014). Nonequilibrium water dynamics in the rhizosphere: How mucilage affects water flow in soils. Water Resour Res.

[CR23] Landl M, Huber K, Schnepf A, Vanderborght J, Javaux M, Bengough AG, Vereecken H (2017). A new model for root growth in soil with macropores. Plant Soil.

[CR24] Malamy JE (2005). Intrinsic and environmental response pathways that regulate root system architecture. Plant Cell Environ.

[CR25] Marco K, Bougoure JJ, Nico PS, Pett-Ridge J, Weber PK, Kleber M (2015). Mineral protection of soil carbon counteracted by root exudates. Nat Climate Change.

[CR26] Materechera SA, Dexter AR, Alston AM (1992). Formation of aggregates by plant-roots in homogenized soils. Plant Soil.

[CR27] Mirreh HF, Ketcheson JW (1973) Influence of soil water matric potential and resistance to penetration on corn root elongation. Can J Soil Sci 53(4):383–388

[CR28] Misra RK, Dexter AR, Alston AM (1986). Maximum axial and radial growth pressures of plant roots. Plant Soil.

[CR29] Mooney SJ, Pridmore TP, Helliwell J, Bennett MJ (2012). Developing X-ray computed tomography to non-invasively image 3-D root systems architecture in soil. Plant Soil.

[CR30] Morel JL, Habib L, Plantureux S, Guckert A (1991). Influence of maize root mucilage on soil aggregate stability. Plant Soil.

[CR31] Moradi AB, Carminati A, Lamparter A, Woche SK, Bachmann J, Vetterlein D, Vogel H-J, Oswald SE (2012) Is the rhizosphere temporarily water repellent? Vadose Zone J. 10.2136/vzj2011.0120

[CR32] Naveed M, Brown LK, Raffan AC, George TS, Bengough AG, Roose T, Sinclair I, Koebernick N, Cooper L, Hallett PD (2017) Plant exudates can either stabilise or weaken soils depending on species, origin and time. Eur J Soil Sci 68 (in press) 10.1111/ejss.12487 PMC5726377 29263712

[CR33] Paterson E, Gebbing T, Abel C, Sim A, Telfer G (2007). Rhizodeposition shapes rhizosphere microbial community structure in organic soil. New Phytol.

[CR34] Peng X, Hallett PD, Zhang B, Horn R (2011). Physical response of rigid and non-rigid soils to analogues of biological exudates. Eur J Soil Sci.

[CR35] Read DB, Gregory PJ (1997). Surface tension and viscosity of axenic maize and lupin root mucilages. New Phytol.

[CR36] Reszkowska A, Krümmelbein J, Peth S, Horn R, Zhao Y, Gan L (2011). Influence of grazing on hydraulic and mechanical properties of steppe soils under different vegetation type in Inner Mongolia. China Plant Soil.

[CR37] Schmidt S, Bengough AG, Gregory PJ, Grinev DV, Otten W (2012). Estimating root-soil contact from 3D X-ray microtomographs. Eur J Soil Sci.

[CR38] Stock O, Downes NK (2008). Effects of additions of organic matter on the penetration resistance of glacial till for the entire water tension range. Soil Till Res.

[CR39] Tarchitzky J, Chen Y (2002). Polysaccharides and pH effects on sodium montmorillonite: Flocculation, dispersion, and rheological properties. Soil Sci.

[CR40] Uteau D, Pagenkemper SK, Peth S, Horn R (2013). Root and time dependent soil structure formation and its influence on gas transport in the subsoil. Soil Till Res.

[CR41] Wang X, Tang C, Guppy CN, Sale PWG (2008). Phosphorus acquisition characteristics of cotton (*Gossypium hirsutum* L.), wheat (*Triticum aestivum* L.) and white lupin (*Lupinus albus* L.) under P deficient conditions. Plant Soil.

[CR42] Watt M, Hugenholtz P, White R, Vinall K (2006). Numbers and locations of native bacteria on field-grown wheat roots quantified by fluorescence insitu hybridization (FISH). Environ Microbiol.

[CR43] Zarebanadkouki M, Carminati A (2014). Reduced root water uptake after drying and rewetting. J Plant Nutr Soil Sci.

[CR44] Zhang B, Hallett PD, Zhang G (2008). Increase in the fracture toughness and bond energy of clay by a root exudate. Eur J Soil Sci.

[CR45] Zhang B, Horn R, Hallett PD (2005). Mechanical resilience of degraded soil amended with organic matter. Soil Sci Soc Am J.

